# Adaptation of red blood cell lysis represents a fundamental breakthrough that improves the sensitivity of *Salmonella* detection in blood

**DOI:** 10.1111/jam.12769

**Published:** 2015-03-12

**Authors:** MA Boyd, SM Tennant, JH Melendez, D Toema, JE Galen, CD Geddes, MM Levine

**Affiliations:** 1 Center for Vaccine Development, University of Maryland BaltimoreBaltimore, MD, USA; 2 Department of Pediatrics, University of Maryland BaltimoreBaltimore, MD, USA; 3 Department of Medicine, University of Maryland BaltimoreBaltimore, MD, USA; 4 Institute of Fluorescence and Department of Chemistry and Biochemistry, University of Maryland Baltimore CountyBaltimore, MD, USA

**Keywords:** blood, detection, diagnostic, invasive, PCR, *Salmonella*, sensitive, typhoid

## Abstract

**Aims:**

Isolation of *Salmonella* Typhi from blood culture is the standard diagnostic for confirming typhoid fever but it is unavailable in many developing countries. We previously described a Microwave Accelerated Metal Enhanced Fluorescence (MAMEF)-based assay to detect *Salmonella* in medium. Attempts to detect *Salmonella* in blood were unsuccessful, presumably due to the interference of erythrocytes. The objective of this study was to evaluate various blood treatment methods that could be used prior to PCR, real-time PCR or MAMEF to increase sensitivity of detection of *Salmonella*.

**Methods and Results:**

We tested ammonium chloride and erythrocyte lysis buffer, water, Lymphocyte Separation Medium, BD Vacutainer® CPT™ Tubes and dextran. Erythrocyte lysis buffer was the best isolation method as it is fast, inexpensive and works with either fresh or stored blood. The sensitivity of PCR- and real-time PCR detection of *Salmonella* in spiked blood was improved when whole blood was first lysed using erythrocyte lysis buffer prior to DNA extraction. Removal of erythrocytes and clotting factors also enabled reproducible lysis of *Salmonella* and fragmentation of DNA, which are necessary for MAMEF sensing.

**Conclusions:**

Use of the erythrocyte lysis procedure prior to DNA extraction has enabled improved sensitivity of *Salmonella* detection by PCR and real-time PCR and has allowed lysis and fragmentation of *Salmonella* using microwave radiation (for future detection by MAMEF).

**Significance and Impact of the Study:**

Adaptation of the blood lysis method represents a fundamental breakthrough that improves the sensitivity of DNA-based detection of *Salmonella* in blood.

## Introduction

*Salmonella enterica* serovars Typhi and Paratyphi A and B cause enteric fever, febrile illnesses that cannot be clinically distinguished from one another and which are difficult to differentiate from other causes of febrile illness (Crump 2012). Developing improved and rapid tests to diagnose these infections is considered one of the highest priorities to achieve control of enteric fever at the individual and population levels. A sensitive, specific, rapid, robust, affordable test is needed that can differentiate febrile patients with treatable enteric fever infections from those who have fever caused by other infectious agents such as dengue, *Plasmodium* or other bacteria. Successful diagnosis of enteric fever patients in a district or region can influence decisions to implement currently licensed typhoid vaccines or future typhoid and paratyphoid A vaccines that are under development (Crump *et al*. 2003, 2004; Levine 2006; DeRoeck *et al*. 2007; Crump and Mintz 2010; Buckle *et al*. 2012; Herrick *et al*. 2012; Lozano *et al*. 2012; Martin 2012). In sub-Saharan Africa, nontyphoidal *Salmonella* (NTS) serovars, in particular, *S*. Typhimurium and *S*. Enteritidis, are cardinal causes of invasive bacterial disease (sepsis, meningitis) in young children and HIV-infected adults and diagnostic tests are needed to identify these infections and to differentiate them from enteric fever (Graham *et al*. 2000; Feasey *et al*. 2012; Okoro *et al*. 2012; MacLennan and Levine 2013).

The current gold standard method for identifying invasive *Salmonella* is their detection by culture of aspirated bone marrow. However, collecting bone marrow as a routine diagnostic is clearly impractical. Accordingly, culture of blood provides a logistically much simpler diagnostic test. Nevertheless, it can take several days to detect and determine the serovar of *Salmonella* in blood or bone marrow (Reisner and Woods 1999; Durmaz *et al*. 2003). Importantly, in developing countries, few health care facilities in low resource settings have the laboratory infrastructure, skilled personnel or budgets required to perform blood or bone marrow cultures. Thus, there is a pressing need for a simpler, more rapid and affordable means to identify invasive *Salmonella* infections to guide therapy in individual patients and to provide epidemiologic information on the endemic circulation of specific serovars in the community (Feasey *et al*. 2012), as well as in outbreak situations (Neil *et al*. 2012).

Polymerase chain reaction (PCR) with gel-based identification of amplicons is potentially more sensitive and rapid than blood culture and reportedly detects as few as 10 CFU ml^−1^ of *Salmonella* in blood (Sanchez-Jimenez and Cardona-Castro 2004). Quantitative real-time PCR (qPCR) has been shown to detect 100–200 organisms per ml of blood (Nga *et al*. 2010). While these technologies, as reported so far, are promising, the reported limits of detection are deemed to be insufficiently sensitive, as the median *S*. Typhi count in the blood of Vietnamese children with suspected enteric fever was 1 CFU ml^−1^ (range <0·3–387 CFU ml^−1^) (Wain *et al*. 1998). Similarly, low concentrations of NTS in blood have been demonstrated by Gordon *et al*. (2010).

Microwave-accelerated metal-enhanced fluorescence (MAMEF) is an ultra-fast, highly sensitive DNA hybridization-based methodology that has been adapted to allow more sensitive detection of *Salmonella* (Tennant *et al*. 2011). This technology integrates metal-enhanced fluorescence using metallic nanoparticle substrates for amplification of fluorescence signatures with low power microwave heating to kinetically accelerate bio-molecular recognition events between basic DNA probes (Aslan *et al*. 2007). MAMEF has been adapted by Tennant *et al*. (2011) to detect the origin of replication (*oriC*) locus that is conserved among all *Salmonella* serovars. Following microwave-accelerated lysis, released and fragmented bacterial DNA is hybridized to a molecular probe, allowing detection of as low as 1 CFU of *Salmonella* in 1 ml of bacteriologic medium in <30 s. The MAMEF *Salmonella* assay is specific and does not detect other bacterial species commonly isolated from blood (Tennant *et al*. 2011).

Although MAMEF reliably detects *Salmonella* in culture medium, initial attempts to detect bacteria in diluted whole blood following lysis were not reproducible due to congealing of erythrocytes and clotting factors during microwave heating. Recognizing that about 60% of *Salmonella* in the blood of an infected individual reside in the mononuclear cells and that direct plating of buffy coat yielded sensitivity comparable to blood culture (Wain *et al*. 1998), we undertook to develop a method that isolates white blood cells for testing by PCR or future MAMEF.

## Materials and methods

### Bacterial strains, blood and ethics statement

Attenuated *Salmonella* Enteritidis strain CVD 1940 (pGEN206) (Tennant *et al*. 2011) and *S*. Typhi live oral vaccine strain CVD 909 (Δ*aroC* Δ*aroD* Δ*htrA* P*tac-tviA)* (Hone *et al*. 1991; Levine *et al*. 1996; Wang *et al*. 2000) were used to safely spike blood. Non-*Salmonella* strains used to determine specificity included *Escherichia coli* Bort, *Pseudomonas aeruginosa* PA01 and *Klebsiella pneumoniae* B5055 from collections at the Center for Vaccine Development (CVD). *Salmonella* spp., *E. coli*, *P. aeruginosa* and *K. pneumoniae* were grown in HS bacteriological medium (5 g sodium chloride, 10 g soytone (Teknova, Hollister, CA), 5 g Hy Yest 412 (Sigma Aldrich, St. Louis, MO) in 1 l distilled water) at 37°C. Media were supplemented with guanine (0·001% w/v) and 50 *μ*g ml^−1^ carbenicillin for growth of CVD 1940(pGEN206); culture medium for CVD 909 was supplemented with 2, 3-dihydroxybenzoate (DHB) (0·0001% w/v). Whole human blood with sodium heparin anticoagulant was purchased from Innovative Research (Novi, MI). For the buffy coat experiments, fresh blood was obtained from volunteers under the approval of the University of Maryland, Baltimore Institutional Review Board and used within 8 h. All blood donors provided written informed consent.

### *Salmonella* uptake assay

7 × 10^3^ CFU of *S*. Enteritidis strain CVD 1940 (pGEN206) were inoculated into 2 ml of whole blood and the buffy coat was separated by mixing the blood with 2 ml of 1× PBS, layering carefully onto lymphocyte separation medium (Corning cellgro^**®**^, Manassas, VA) and centrifuging at 400 ***g*** for 10 min. The buffy coat was removed and lysed with a final concentration of 1% Triton X-100 (Sigma Aldrich, St. Louis, MO). Viable counts were performed by mixing the lysed buffy coat with 20 ml of molten HS-agar supplemented with guanine (0·001% w/v) and 50 *μ*g ml^−1^ carbenicillin. Colonies were counted after 24-h incubation at 37°C and the percentage of intracellular bacteria recovered was determined in relation to the initial inoculum.

### Preparation of blood

Six techniques to separate white blood cells from erythrocytes were evaluated, including erythrocyte lysis buffer, ammonium chloride lysis buffer, distilled water, BD Vacutainer® CPT™ tubes, dextran, and lymphocyte separation medium, as described below. Following erythrocyte lysis, white blood cell pellets were suspended in various diluents (1× TE buffer, 1× PBS, 0·05× PBS, animal product-free LB Lennox medium (APF-LB; Athena Environmental Sciences, Baltimore, MD) and 1× TE buffer plus 5 g l^−1^ of NaCl), as necessary.

#### Erythrocyte and ammonium chloride lysis buffer

Two ml of whole human blood were mixed with 18 ml of 1× erythrocyte lysis buffer diluted from a 10× stock (82·6 g ammonium chloride, 0·37 g EDTA and 10 g potassium phosphate in 1 l distilled water) or ammonium chloride buffer alone (8·26 g ammonium chloride in 1 l distilled water). Each sample was inverted 10 times, incubated at room temperature for 2 min and centrifuged for 5 min at 400 ***g*** using a swing bucket rotor followed by removal of the supernatant, thus removing the erythrocyte debris and leaving behind a pellet consisting of white blood cells and a small amount of erythrocytes. The pellet was resuspended in 20 ml of fresh erythrocyte lysis buffer (ammonium chloride alone or 1× erythrocyte lysis buffer), inverted five times and centrifuged again to ensure complete lysis of erythrocytes. The supernatant was removed leaving behind a white blood cell pellet.

#### Water

Two millilitres of whole blood were mixed with 18 ml of distilled water. The sample was inverted 10 times, incubated at room temperature for 2 min and centrifuged for 5 min at 400 ***g***, thus removing the erythrocyte cell debris and leaving behind a white blood cell pellet. The pellet was resuspended in 20 ml water, inverted five times and centrifuged again to ensure complete erythrocyte lysis. The supernatant was removed leaving behind a white blood cell pellet.

#### BD Vacutainer® CPT™ (Becton Dickinson, Franklin Lakes, NJ)

Buffy coat was obtained by drawing 8 ml of whole blood into a BD Vacutainer® CPT™ cell preparation tube containing sodium heparin and centrifuged in a swing bucket centrifuge at 1500 ***g*** for 15 min. The buffy coat layer was transferred to a fresh tube, resuspended and washed in 1× PBS and centrifuged at 16 000 ***g*** for 3 min to remove the PBS.

#### Dextran

Buffy coat was obtained by mixing 2 ml of fresh whole blood with 2 ml of 2% dextran (Sigma Aldrich) in 0·9% normal saline containing 25 mmol l^−1^ sodium citrate and incubated for 30 min at room temperature (Boyum 1964). The buffy coat, which was the upper layer, was washed twice with 1× PBS in a fresh tube and centrifuged at 16 000 ***g*** for 3 min to remove the erythrocytes and pellet the white blood cells.

#### Lymphocyte separation medium

Buffy coat was obtained by mixing 2 ml of fresh whole blood with 2 ml of 1× PBS and layering onto 2 ml lymphocyte separation medium (Corning cellgro^**®**^). The mixture was centrifuged at 400 ***g*** using a swing bucket rotor for 5 min to separate the buffy coat from the erythrocytes and plasma.

### Detection of *Salmonella* DNA by gel-based PCR

#### Extraction of DNA from spiked whole blood

DNA was extracted from 2 ml of whole blood spiked with 4·4 × 10^3^ CFU, 4·4 × 10^4^ CFU, 4·4 × 10^5^ CFU or 4·4 × 10^6^ CFU of CVD 909 using the QIAamp DNA Blood Midi Kit (Qiagen, Valencia, CA) according to the manufacturer's instructions. The purified DNA was eluted in 300 *μ*l of nuclease-free water.

#### Extraction of DNA from spiked white blood cells

Two millilitres of whole blood was lysed using erythrocyte lysis buffer as described above. The white blood cell pellets were resuspended in 200 *μ*l PBS and subsequently spiked with 4·4 × 10^3^ CFU, 4·4 × 10^4^ CFU, 4·4 × 10^5^ CFU or 4·4 × 10^6^ CFU of CVD 909 and DNA extraction was performed using the QIAamp DNA Blood Mini Kit (Qiagen) according to the manufacturer's instructions. The purified DNA was eluted in 100 *μ*l of nuclease-free water.

#### PCR

PCR amplification was performed in 1× PCR buffer containing 1·5 mmol l^−1^ MgCl_2_, 2·5 U Green *Taq* DNA polymerase (Genscript, Piscataway, NJ), 0·2 mmol l^−1^ dNTP and primers oriCF (5′- TTATTAGGATCGCGCCAGGC-3′) and oriCR (5′- AAAGAATAACCGTTGTTCAC-3′) which detect all *Salmonella* at a final concentration of 0·5 *μ*mol l^−1^ and combined with 5 *μ*l of extracted DNA in a reaction volume of 50 *μ*l in a T100™ Thermal Cycler (Bio-Rad laboratories, Hercules, CA) (Widjojoatmodjo *et al*. 1991; Levy *et al*. 2008). Cycling parameters were as follows: 94°C for 3 min, followed by 30 cycles at 94°C for 30 s, 55°C for 30 s and 72°C for 30 s with a final extension of 72°C for 5 min. The primers described above yielded an amplicon size of 163 bp. PCR products were separated on 2% (w/v) agarose gels, stained with ethidium bromide and visualized using a UV transilluminator.

### Detection of *Salmonella* DNA by real-time PCR

#### Extraction of DNA from spiked whole blood

DNA was extracted from 2 ml of whole blood spiked with 4, 41, 410 CFU or 4100 CFU of CVD 909 using the QIAamp DNA Blood Midi Kit (Qiagen) according to the manufacturer's instructions. Concentrations of CVD 909 were obtained by serial dilution in HS bacteriological medium. The purified DNA was eluted in 300 *μ*l of nuclease-free water.

#### Extraction of DNA from spiked white blood cells

Two millilitres of whole blood was lysed using erythrocyte lysis buffer as described above. The white blood cell pellets were resuspended in 200 *μ*l PBS and subsequently spiked with three ten-fold dilution series of CVD 909 (ranging from 0·3 CFU to 6 × 10^5^ CFU). DNA was extracted using the QIAamp DNA Blood Mini Kit (Qiagen) according to the manufacturer's instructions. Viable counts of CVD 909 were obtained by serial dilution on HS-agar plates. The purified DNA was eluted in 50 *μ*l of nuclease-free water or AE buffer from the QIAamp kit.

#### PCR

Primers used for real-time PCR targeted STY0201, a *S*. Typhi putative fimbrial-like adhesion protein and were taken from Nga *et al*. (Nga *et al*. 2010). The primer and probe sequences are as follows: ST-Frt, 5′ CGCGAAGTCAGAGTCGACATAG 3′; ST-Rrt, 5′ AAGACCTCAACGCCGATCAC 3′; and, ST- Probe, 5′ FAM-CATTTGTTCTGGAGCAGGCTGACGG-TAMRA 3′. Reactions were performed using TaqMan® Fast Universal PCR Master Mix (2×), No AmpErase® UNG (Life Technologies, Grand Island, NY), combined with 5 *μ*l template DNA, 0·9 *μ*mol l^−1^ forward and reverse primers and 0·25 *μ*mol l^−1^ probe in a 20 *μ*l reaction volume. Real-time PCR amplification was performed on an ABI Fast 7500DX instrument (Life Technologies). Cycling parameters were as follows: initial denaturation of 95°C for 20 s, followed by 40 cycles of denaturation at 95°C for 3 s, annealing and extension at 60°C for 30 s.

### Lysis of *Salmonella* by microwave radiation

In order to focus microwaves, gold bowtie lysis triangles were deposited on glass slides as described previously (Tennant *et al*. 2011). A single self-adhesive silicon isolator (33 mm diameter) (Stockwell Elastomerics, Philadelphia, PA) was placed over the gold bow-tie triangles to create a 5-ml lysing chamber. To optimize the lysing procedure which both lyses bacteria and breaks the DNA into small fragments, white blood cells were resuspended in 1 ml of various diluents following red blood cell lysis. Overnight cultures of bacteria (10^9^ CFU ml^−1^) were diluted to the desired concentration in PBS and 10 *μ*l of bacteria were added to the white blood cell suspension. The bacteria-spiked white blood cell samples were then heated in a Frigidaire microwave (Model No. FFCM0934LB; 900 watts) for 25 s on 30% power. In order to determine the optimal media for microwave lysis, lysis in 1 ml of water was compared to the same volume of 1× TE buffer, 1× PBS, 0·05× PBS, APF-LB and 1× TE buffer plus 5 g l^−1^ of NaCl. A cracking scale for the gold lysis bow-tie structures after exposure to microwave irradiation was determined on a scale of 1–5, with 1 representing small cracks and 5 representing complete disintegration of the gold bow ties. For visualization of DNA fragments on a gel, 1 ml overnight bacterial culture containing 10^9^ CFU *Salmonella* was pelleted, resuspended in 1 ml TE plus 5 g l^−1^ NaCl and added to white blood cells separated from 2 ml whole blood by erythrocyte blood cell lysis buffer. The suspension was microwave lysed and DNA was ethanol precipitated with 0·1× volume of 3 mol l^−1^ sodium acetate pH 5·2 and 2× volume of prechilled molecular grade ethanol. DNA was pelleted, washed with 70% ethanol, air-dried and resuspended in 30 *μ*l of TE and the entire volume was electrophoresed on a 2% agarose gel. DNA fragments were visualized by staining with ethidium bromide to identify double stranded DNA or SYBR® Gold (Invitrogen, Eugene, OR) to identify single stranded DNA. DNA fragment size was verified with the Agilent 2100 Bioanalyzer (Agilent Technologies, Santa Clara, CA).

## Results

### *Salmonella* uptake by white blood cells

**Figure 1:**
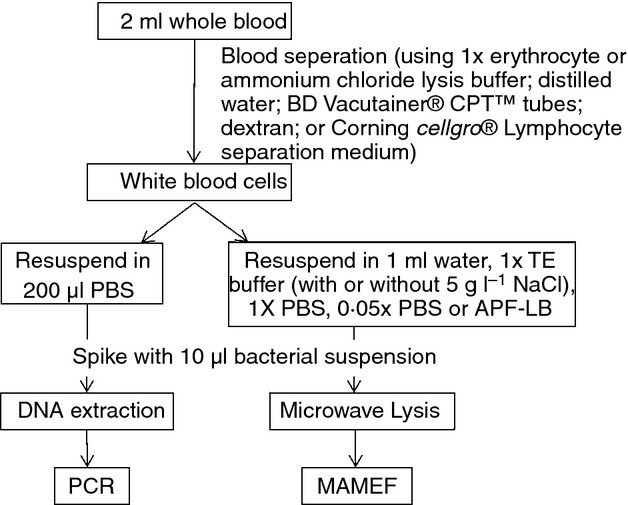
Flow chart of procedure for blood lysis and subsequent DNA detection by PCR and MAMEF.

In patients with typhoid fever the majority of *S*. Typhi reside in mononuclear phagocytes. We therefore conducted a *Salmonella* uptake assay using fresh whole blood and separating the white blood cells to assess uptake in a laboratory setting. When whole blood was removed from a healthy donor and inoculated with 7 × 10^3^ CFU of *Salmonella*, an average of only 1·4% (range of 1·2–1·6%) was found to be taken up by the mononuclear cells, results which are corroborated in previous studies by Rubin *et al*. (1990). Hence, to account for this limitation in the laboratory setting, *Salmonella* were added to isolated white blood cells after the processing of blood by the various separation techniques that we tested (Fig.1).

### Blood lysis

**Figure 2:**
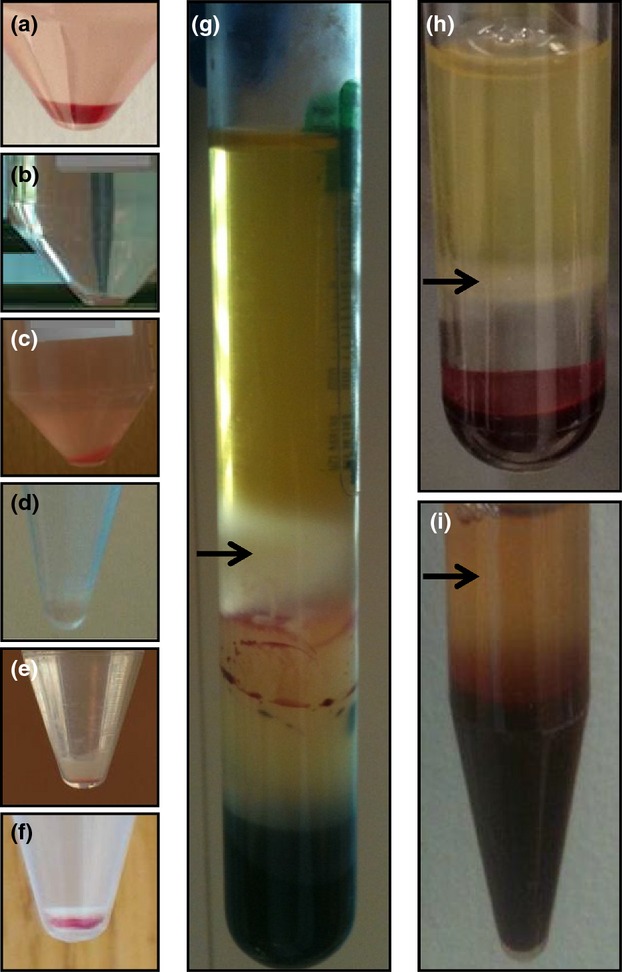
Blood lysis. White blood cell pellet obtained when whole blood is processed by ammonium chloride (a), erythrocyte lysis buffer (b), water (c), BD Vacutainer® CPT™ Tube (d), dextran (e) and lymphocyte separation medium (f). Buffy coat (arrows) separated by BD Vacutainer® CPT™ Tube (g), lymphocyte separation medium (h) and dextran (i).

We tested six techniques for separating white blood cells from whole blood. Three methods involved whole blood lysis and the remaining three methods involved buffy coat separation. Of the three whole blood lysis techniques (either ammonium chloride alone, 1× erythrocyte lysis buffer or water), 1× erythrocyte lysis buffer removed erythrocytes most effectively, and yielded the purest sample of white blood cells (Fig.2). When this technique was applied to 2 ml of whole blood spiked with 10^3^ CFU ml^−1^ of *Salmonella*, 40% of the extracellular bacteria were recovered from the pellet after centrifugation. Of the three buffy coat separation techniques tested, the white blood cells obtained from the BD Vacutainer® CPT™ tube achieved the most complete separation of white blood cells followed by the lymphocyte separation medium and then dextran (Fig.2). However, the BD Vacutainer® CPT™ tube was the most expensive of the separation techniques tested here (Table 1). Erythrocyte lysis buffer and dextran were found to be the two best methods as they yielded the purest samples of white blood cells (i.e. removed erythrocytes most effectively), utilized inexpensive reagents, involved fast processing and allowed both intracellular and extracellular *Salmonella* to be isolated.

**Table 1:** Comparison of blood separation methods

Blood component	Blood separation method	Cheap reagents	Fast*	Obtain intracellular and extracellular bacteria	Large equipment†	Small equipment‡
Whole Blood	Erythrocyte or ammonium chloride lysis buffer	Y§	Y	Y	Y	N
	Water	Y	Y	Y	Y	N
Buffy Coat	Dextran	Y	Y	Y	N	Y
	Lymphocyte separation medium	N	N	N	Y	Y
	BD Vacutainer® CPT™	N	Y	N	N	Y

* Processing time is 45 min for 10 samples.

† Requires bench top centrifuge with swing bucket rotor.

‡ Requires microcentrifuge.

§ Y, Yes; N, No.

### Gel-based PCR detection of *Salmonella*

**Figure 3:**
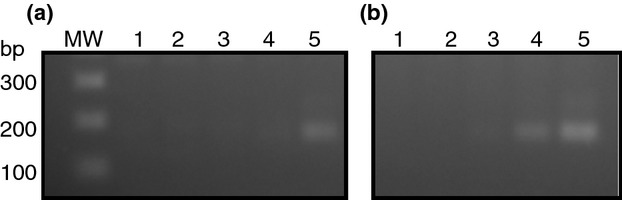
Gel-based PCR detection of *Salmonella* DNA. Detection of *oriC* by PCR on DNA extracted from spiked whole blood or pretreated blood. (a) DNA extracted from whole blood (using a QIAmp Midi blood kit). (b) DNA extracted from lysed blood (erythrocyte lysis followed by QIAmp Mini blood kit). Lane MW, 1 Kb plus DNA ladder (Invitrogen). Lanes 1, blood only; 2, blood spiked with 4·4 × 10^3^ CFU CVD 909; 3, blood spiked with 4·4 × 10^4^ CFU CVD 909; 4, blood spiked with 4·4 × 10^5^ CFU CVD 909; and 5, blood spiked with 4·4 × 10^6^ CFU CVD 909.

To determine whether sensitivity of detection of *Salmonella* in blood could be improved by targeting white blood cells instead of using whole blood, we used conventional gel-based PCR. We found that when the QIAamp Mini blood kit was used to extract DNA from spiked white blood cells (separated from 2 ml whole blood using 1× erythrocyte buffer), we could detect 4·4 × 10^5^ and 4·4 × 10^6^ CFU of *S*. Typhi CVD 909 by PCR. In contrast, when the QIAamp Midi blood kit was used to extract DNA from 2 ml spiked whole blood, only the 4·4 × 10^6^ CFU sample was detected (Fig.3). 4·4 × 10^3^ CFU and 4·4 × 10^4^ CFU *Salmonella* was not detected using either extraction method.

### Real-time PCR detection of *Salmonella*

**Figure 4:**
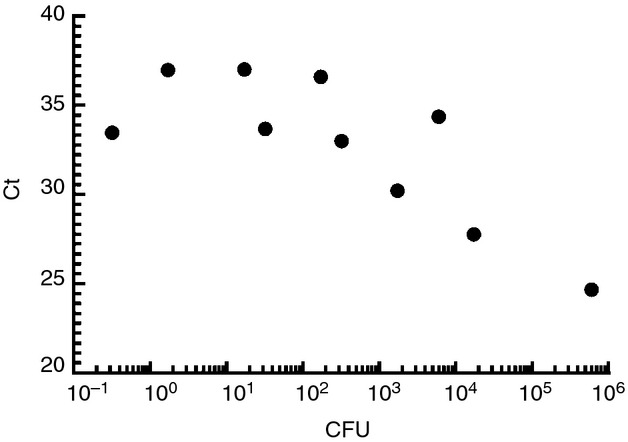
Real-time PCR detection of *Salmonella* DNA. Two millilitres of whole blood was lysed using erythrocyte lysis buffer and spiked with the shown CFU of *Salmonella* Typhi CVD 909. DNA was extracted using a QIAamp Mini blood kit and *S.* Typhi was detected by qPCR.

Following the promising results using gel-based PCR, we determined whether combining RBC lysis with a mini QIAamp kit could also lead to improved detection by real-time PCR. First, we spiked whole blood with various concentrations of *S*. Typhi and performed qPCR as described by Nga *et al*. (2010) who showed a detection limit for this assay of 100–200 organisms per ml blood when DNA was extracted using a QIAamp blood midi kit. In our hands, using the same procedure, we were able to detect 41 CFU suspended in 2 ml blood (Table 2). We found that when the QIAamp Mini blood kit was used to extract DNA from spiked white blood cells (separated from 2 ml whole blood using 1× erythrocyte buffer), we could detect as low as 0·3 CFU by real-time PCR (Fig.4). As expected, we observed an inverse relationship between CFU and cycle threshold (*C*_t_) values.

**Table 2:** Real-time PCR detection of *Salmonella* Typhi in 2 ml blood using DNA extracted by a QIAamp DNA midi kit as per Nga *et al*. (2010)

CFU per 2 ml blood	*C*_t_ value	Standard deviation	No. of replicates positive/Total no. of replicates tested
4120	33·52	0·3	3/3
412	37·26	1·14	3/3
41	38·27	0·46	3/3
4	ND	ND	0/3

ND, Not detected.

### Microwave lysis of *Salmonella* and fragmentation of DNA

Previous attempts to detect *Salmonella* in whole blood by MAMEF were unsuccessful due to congealing of erythrocytes and clotting factors under microwave heating (results not shown). The role of clotting factors in congealing was supported by the fact that when plasma and serum were microwave lysed, samples also congealed under exposure to microwave heat (results not shown).

We took advantage of the fact that approx. 60% of *Salmonella* are intracellular and focused our attempts on harvesting and lysing white blood cells. We tested microwave lysis of white blood cells spiked with *Salmonella* and suspended in various diluents. When approx. 1000 CFU of bacteria were serially diluted in PBS down to 0·1 CFU, added to purified white blood cells, resuspended in 1 ml TE plus 5 g l^−1^ NaCl and microwave lysed, 100% lysis was observed reproducibly in spiked samples containing 100 CFU or more as determined by pre- and postlysis viable counts (>10 samples tested per bacterial dilution). As a result of complete removal of erythrocytes using 1× erythrocyte buffer, we were able to avoid congealing under microwave heat.

**Figure 5:**
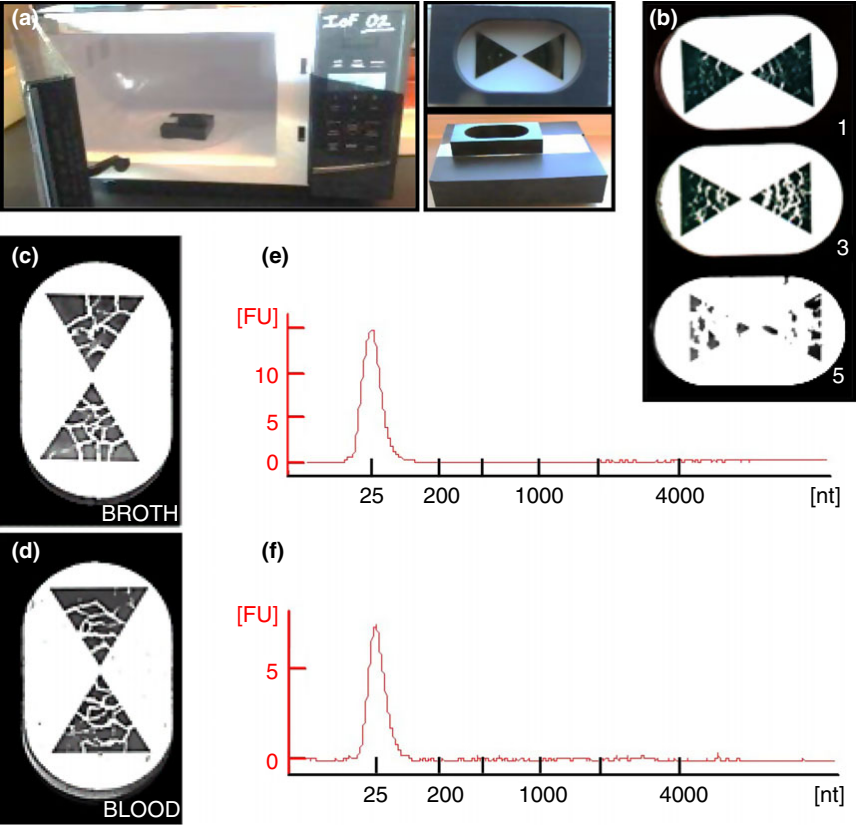
Microwave lysis of *Salmonella*. (a) Gold lysing triangles with bowtie configuration and silicone holder in a microwave. (b) Increasing gold cracking with increasing salt concentration (Gold cracking scale 1–5: 1, small cracks; 5, completely cracked). (c) Gold lysing triangle showing extent of cracking when *Salmonella* is lysed in broth. (d) Gold lysing triangle showing extent of cracking when *Salmonella* is lysed in blood. (e–f) Fragment size of DNA released from *Salmonella* lysed in broth and blood, respectively, using an Agilent 2100 Bioanalyzer.

**Figure 6:**
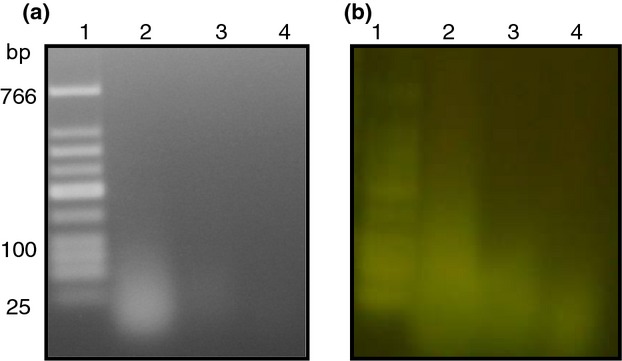
DNase and RNase treatment of *Salmonella*. (a) Ethidium bromide, and (b) SYBR gold stained 2% agarose gels. Lane 1, Low Molecular Weight DNA ladder (New England Biolabs, Ipswich, MA); Lane 2, nucleic acid from lysed whole blood released from *Salmonella* in blood (10^9^ CFU ml^−1^) after microwave lysis; Lane 3, nucleic acid from lysed whole blood containing 10^9^ CFU ml^−1^ *Salmonella* and treated with RNase; Lane 4, nucleic acid from lysed whole blood containing 10^9^ CFU ml^−1^ *Salmonella* and treated with DNase.

We also observed that when too little salt was present during microwave lysis, DNA release and fragmentation was suboptimal (as evidenced by a DNA smear on an agarose gel with a peak band of approx. 100 bp compared to a single approx. 25 bp band when fragmentation is complete). On the other hand, when too much salt was present, the sample reached boiling temperature too rapidly and the sample boiled over in the microwave (Table 3). When the white blood cells were resuspended in water alone (0 mol l^−1^ NaCl), the peak fragment size was approx. 100 bp. Similarly, when the white blood cells were resuspended in 0·05× PBS, which contains approx. 6 mmol l^−1^ sodium chloride, DNA fragments of about 100 bp were also observed (Table 3). In contrast, samples containing white blood cells resuspended in 1× PBS (0·15 mol l^−1^ sodium chloride) boiled over in the microwave due to overheating at these high salt concentrations and sample was lost in the microwave cavity. Optimal conditions for microwave lysis were ultimately identified as white blood cells suspended in APF-LB (previously used for microwave lysis of *Salmonella* as described by Tennant *et al*. (2011)) or 1× TE buffer plus 5 g l^−1^ NaCl (which both contain 80 mmol l^−1^ NaCl). Eventually, we were able to estimate the effectiveness of microwave lysing and DNA release by visually inspecting the cracking of the gold bow tie after microwave lysis with more cracking signalling maximal DNA release and fragmentation (Fig.5b). Lysis was optimized by adjusting the microwave power and time until maximal DNA release and fragmentation to approx. 25 bp was achieved at 30% power and 25 s from white blood cells suspended in 1 ml 1× TE buffer plus 5 g l^−1^ NaCl and 10^9^ CFU of CVD 909. In order to confirm that the fragment size achieved from microwave lysed white blood cells spiked with *Salmonella* (and resuspended in medium containing 80 mmol l^−1^ NaCl) was the same as was previously optimized for broth samples as described by Tennant *et al*. (2011), side by side comparison of lysis of *Salmonella* in APF-LB *vs* microwave lysis of spiked white blood cells was performed. We examined gold cracking and also verified fragment sizes using the Agilent 2100 Bioanalyzer (Fig.5c–f). As shown in Fig.6, SYBR® Gold staining revealed that the DNA was mostly single stranded. Treatment of samples with DNase or RNase suggested that the nucleic acid released was mostly DNA and not RNA (Fig.6). As a result of complete removal of erythrocytes, we were able to avoid congealing under microwave heat and enable efficient lysis of samples.

**Table 3:** Salt concentration-dependent fragmentation of DNA and gold cracking produced by microwave lysis

Lysis solution	NaCl concentration, mol l^−1^	Gold cracking*	Average fragment size†
Water	0	3	100 bp
TE	0	3	100 bp
0·05× PBS	0·006	3	100 bp
APF-LB	0·08	4·5	25 bp
TE + 5 g l^−1^ NaCl	0·08	4·5	25 bp
1× PBS	0·136	4·5	Sample boiled over

* Gold cracking scale 1–5: 1, small cracks; 5, completely cracked.

† DNA fragment size determined by visualizing SYBR Gold- and ethidium bromide-stained agarose gels or using an Agilent 2100 Bioanalyzer after microwave lysis at 30% power, 25 s.

## Discussion

We have previously shown that *Salmonella* suspended in bacteriological medium can be successfully lysed using microwaves, allowing highly sensitive detection of *Salmonella* DNA by MAMEF (Tennant *et al*. 2011). However, when we attempted detection of a synthetic *oriC* oligonucleotide suspended in blood, MAMEF was not able to detect a fluorescent signal. When the spiked blood sample was diluted with an equal volume of PBS, a fluorescent signal was detected, presumably due to a reduction in the viscosity of blood. After repeated experiments using diluted blood, we found that sometimes the blood would congeal during microwave heating, which rendered it impossible for the fluorescent signal to be detected. We determined that it was necessary to remove the erythrocytes in order to avoid congealing under microwave radiation and to allow the fluorophore to be detected.

Cognizant of the fact that the majority of *Salmonella* organisms in an infected individual reside in the mononuclear cells, we compared two fundamental approaches to obtain the mononuclear cells. In the first approach we treated whole blood by various methods to lyse the erythrocytes and thereupon pelleted the white blood cells. In the alternative approach, we kept the erythrocytes intact, while separating out the mononuclear cell-rich buffy coat layer. Whereas approx. 60% of *S*. Typhi are found within mononuclear cells in a typhoid fever patient, when blood from a healthy human is spiked with *Salmonella in vitro*, only approx. 1–2% of the bacteria are taken up by monocytes. To overcome this experimental limitation, we spiked the white blood cells after treating the blood with various techniques. This provided a means to more accurately compare the efficiency of our various lysis and detection methods.

Each blood treatment method has its advantages and disadvantages (Table 1). It is attractive to have the ability to obtain both extracellular and intracellular *Salmonella* as is possible by treating blood with ammonium chloride, erythrocyte lysis buffer or water. Moreover, these are inexpensive reagents and the methods are practical and fast. However, they suffer one possible limitation for developing country laboratories, namely, the separation of white blood cells and bacteria requires the use of a bench top centrifuge. Even then, while all the intracellular organisms may be recovered, not all the extracellular organisms pellet at the low speed needed for optimal white blood cell separation. Although water is a simple and inexpensive method for lysis, it is less well established and may cause premature lysis of white blood cells and their bacteria; release of DNA earlier than desired may result in loss of DNA when the supernatant is discarded. Overall, erythrocyte lysis buffer was our preferred method of treating whole blood.

For the buffy coat methods, blood must be processed within a 12-h period for optimal separation of the buffy coat. For the lymphocyte separation medium, in addition to requiring a bench top centrifuge, more skilled handling of small volumes of blood is necessary to prevent the disruption of the interface between the buffy coat layer and the erythrocytes. On the other hand, while the BD Vacutainer® CPT™ tube requires a simple and cheap clinical swing bucket centrifuge and can be processed on site, the high cost (∼$9 per tube) makes it too expensive for routine use in many resource poor areas. Among the buffy coat extraction methods, dextran is the most promising because it is easy to perform, inexpensive, easy to handle and requires only an inexpensive, small microcentrifuge. However, it is highly effective only when used with fresh blood.

For utilization in field situations where blood may be collected from a remote site and transported for some hours to a blood processing laboratory, erythrocyte lysis buffer is the preferred method for isolation of white blood cells, as it does not require fresh blood for optimal separation. Additionally, as we showed that we could recover an additional 40% of extracellular *Salmonella* after erythrocyte lysis, it is conceivable that if 1000 CFU of *Salmonella* were present in a sample of blood, 600 CFU (60%) would be recovered from the intracellular portion along with 160 CFU (16%) of the 400 extracellular *Salmonella,* totalling 760 CFU, i.e. 76% of the CFU present in the entire blood sample.

Despite the limitations inherent in each separation method, one major advantage common to all is that any volume of blood may be processed and the white blood cells resuspended in any volume necessary for detection by molecular methods such as MAMEF or PCR or quantitative real-time PCR. This is particularly important in *Salmonella* infection in young and malnourished subjects from whom only small volumes of blood are typically collected to make a laboratory diagnosis.

It is important to stress that testing white blood cells alone from typhoid fever patients does not decrease sensitivity, as has already been demonstrated by Wain *et al*. (1998), where culture of whole blood *vs* buffy coat were equally sensitive. We have shown improved sensitivity using conventional PCR on DNA isolated from spiked white blood cells obtained by erythrocyte lysis and a QIAamp DNA Blood Mini kit compared to DNA from spiked whole blood obtained from a QIAamp DNA Blood Midi kit. Ten-fold less bacteria were detected by gel-based PCR using white blood cells compared to whole blood (4·4 × 10^5^ CFU *vs* 4·4 × 10^6^ CFU). Likewise, when real-time PCR was used, we were able to detect as low as 0·3 CFU *S*. Typhi mixed with white blood cells from 2 ml of blood. In contrast, detection of DNA extracted from whole blood using a midi kit shows a detection limit of more than 40 CFU *S*. Typhi. The increased sensitivity is most likely due to the fact that we could elute the DNA in a smaller volume using the QIAamp DNA Blood Mini kit than the QIAamp DNA Blood Midi kit. As it is not possible to have <1 CFU, we hypothesize that we are also detecting dead bacteria which is an advantage of using molecular methods.

We have also shown that we can effectively lyse *Salmonella* suspended in a buffy coat fraction in preparation for detection by MAMEF. Work is currently underway to determine the level of sensitivity of MAMEF.

We conclude that adaptation of the erythrocyte lysis procedure has enabled improved sensitivity of detection by PCR and real-time PCR following DNA extraction and has allowed lysis and fragmentation of *Salmonella* using microwave radiation (for future detection by MAMEF). We are currently evaluating whether erythrocyte lysis and subsequent detection by real-time PCR is faster and more sensitive than blood culture in a typhoid endemic setting. These blood treatment methodologies may also be applicable for the typhoid/paratyphoid diagnostic assay (TPTest) that detects IgA antibody to *Salmonella* antigens elaborated by mononuclear cells (Khanam *et al*. 2013). Importantly, these methods are economical and can be adapted for use in field studies using molecular methods. They may also be used for diagnosis in hospitals that lack blood culture machines and antisera for identification of *Salmonella,* but which are capable of performing molecular biology procedures.
